# Supplementary material for: Improving AlphaFold2-based protein tertiary structure prediction with MULTICOM in CASP15
Source: Commun Chem. 2023 Sep 7;6:188. doi: 10.1038/s42004-023-00991-6 (PMC10484931; doi:10.1038/s42004-023-00991-6)
Supplement: Supplementary file 2 — Supplementary information [file 42004_2023_991_MOESM2_ESM.pdf]

Supplementary Information

Supplementary Table 1. The performance of the top 20 out of 47 CASP15 server predictors, quantified by the average GDT-TS of the top 1 model submitted by the server predictors for the 68 full-length targets, 31 TBM full-length targets and 37 FM full-length targets (a target is classified as a FM target if one of its domains is FM/TBM or FM domain). NBIS-AF2-standard is the standard AlphaFold2 predictor.

	Server predictors	Avg. GDT-TS on 68 targets	Avg. GDT-TS on 31 TBM targets	Avg. GDT-TS on 37 FM targets
162	UM-TBM	0.7898	0.8821	0.7126
229	Yang-server	0.785	0.872	0.712
475	MULTICOM-refine	0.7543	0.8808	0.6483
35	Manifold-E	0.7508	0.8525	0.6657
86	MULTICOM_qa	0.7484	0.8771	0.6405
158	MULTICOM_deep	0.7474	0.8772	0.6387
120	MULTICOM_egnn	0.7456	0.8759	0.6365
288	DFolding-server	0.7427	0.8584	0.6457
282	GuijunLab-Threader	0.7325	0.8604	0.6254
166	RaptorX	0.7303	0.8564	0.6247
298	MUFold	0.7267	0.8707	0.606
383	server_124	0.7265	0.8589	0.6155
188	GuijunLab-DeepDA	0.7257	0.8643	0.6096
98	GuijunLab-Assembly	0.7241	0.866	0.6051
353	hFold	0.7231	0.851	0.6158
125	UltraFold_Server	0.721	0.8689	0.5972
446	Shennong	0.7173	0.8747	0.5855
466	ColabFold	0.7112	0.8211	0.6191
462	MultiFOLD	0.7074	0.8683	0.5726
270	NBIS-AF2-standard	0.6882	0.7983	0.5959

Supplementary Table 2. The average TM-score of the top 1 model submitted by the top 20 server predictors for all 94 domains, 47 TBM domains and 47 FM and FM/TBM domains.

Predictor ID	Server name	Avg. TM-score on 94 domains	Avg. TM-score on 47 TBM domains	Avg. TM-score on 47 FM or FM/TBM domains
162	UM-TBM	0.8824	0.9253	0.8395
229	Yang-server	0.8763	0.9171	0.8354
475	MULTICOM_refine	0.8332	0.9172	0.7493
120	MULTICOM_egnn	0.8298	0.9086	0.7511
158	MULTICOM_deep	0.8289	0.9098	0.748
35	Manifold-E	0.8289	0.9098	0.7479
86	MULTICOM_qa	0.8286	0.9092	0.7479

166	RaptorX	0.8228	0.9057	0.7398
288	DFolding-server	0.8185	0.8996	0.7374
383	server_124	0.8113	0.9115	0.7111
188	GuijunLab-DeepDA	0.8055	0.9083	0.7027
298	MUFold	0.8047	0.9039	0.7054
282	GuijunLab-Threader	0.8008	0.8953	0.7063
98	GuijunLab-Assembly	0.7988	0.8983	0.6992
125	UltraFold_Server	0.7976	0.9039	0.6912
462	MultiFOLD	0.7954	0.8994	0.6914
446	Shennong	0.7916	0.8952	0.688
353	hFold	0.7837	0.8875	0.68
466	ColabFold	0.7731	0.8138	0.7323
270	NBIS-AF2-standard	0.769	0.8503	0.6876

Supplementary Table 3. The average lDDT of the top 1 model submitted by the top 20 server predictors for all 94 domains, 47 TBM domains and 47 FM and FM/TBM domains.

Predictor ID	Server name	Avg. lDDT on 94 domains	Avg. lDDT on 47 TBM domains	Avg. lDDT on 47 FM or FM/TBM domains
162	UM-TBM	0.8149	0.8533	0.7764
229	Yang-server	0.7942	0.8337	0.7546
475	MULTICOM_refine	0.7934	0.8529	0.7338
120	MULTICOM_egnn	0.7902	0.848	0.7323
158	MULTICOM_deep	0.7901	0.8489	0.7312
86	MULTICOM_qa	0.789	0.8488	0.7292
166	RaptorX	0.7881	0.8491	0.7271
383	server_124	0.778	0.8495	0.7065
288	DFolding-server	0.7769	0.8404	0.7135
298	MUFold	0.7757	0.8477	0.7037
35	Manifold-E	0.774	0.8414	0.7067
98	GuijunLab-Assembly	0.7729	0.8429	0.7029
188	GuijunLab-DeepDA	0.7719	0.8455	0.6984
282	GuijunLab-Threader	0.7699	0.8375	0.7022
125	UltraFold_Server	0.7684	0.8415	0.6952
446	Shennong	0.7681	0.8409	0.6954
462	MultiFOLD	0.767	0.8421	0.6918
353	hFold	0.7558	0.8275	0.684
466	ColabFold	0.747	0.768	0.7259
270	NBIS-AF2-standard	0.7377	0.7929	0.6824

Supplementary Table 4. The average TM-score of the top 1 model submitted by the top 20 server predictors on the 68 full-length targets, 31 TBM full-length targets and 37 FM full-length targets (a target is classified as a FM target if one of its domains is FM/TBM or FM domain).

Predictor ID	Server name	Avg. TM-score on 68 targets	Avg. TM-score on 31 TBM targets	Avg. TM-score on 37 FM targets
162	UM-TBM	0.8454	0.9131	0.7887
229	Yang-server	0.8366	0.9049	0.7794
35	Manifold-E	0.8060	0.8865	0.7386
475	MULTICOM-refine	0.8007	0.9102	0.7090
288	DFolding-server	0.7974	0.8952	0.7155
158	MULTICOM_deep	0.7959	0.9076	0.7023
86	MULTICOM_qa	0.7954	0.9060	0.7027
120	MULTICOM_egnn	0.7936	0.9049	0.7004
282	GuijunLab-Threader	0.7865	0.8931	0.6971
166	RaptorX	0.7816	0.8878	0.6926
383	server_124	0.7771	0.8929	0.6801
298	MUFold	0.7759	0.9043	0.6684
188	GuijunLab-DeepDA	0.7753	0.8953	0.6747
353	hFold	0.7751	0.8873	0.6811
98	GuijunLab-Assembly	0.7734	0.8976	0.6693
125	UltraFold_Server	0.7719	0.9025	0.6624
446	Shennong	0.7629	0.9040	0.6448
466	ColabFold	0.7552	0.8400	0.6842
462	MultiFOLD	0.7550	0.9002	0.6333
270	NBIS-AF2-standard	0.7373	0.8299	0.6598

Supplementary Table 5. The average IDDT of the top 1 model submitted by the top 20 server predictors for the 68 full-length targets, 31 TBM full-length targets and 37 FM full-length targets (a target is classified as a FM target if one of its domains is FM/TBM or FM domain).

Predictor ID	Server name	Avg. IDDT on 68 targets	Avg. IDDT on 31 TBM targets	Avg. IDDT on 37 FM targets
162	UM-TBM	0.8184	0.8587	0.7846
475	MULTICOM-refine	0.8081	0.8583	0.766
120	MULTICOM_egnn	0.8045	0.8568	0.7606
158	MULTICOM_deep	0.804	0.859	0.758
86	MULTICOM_qa	0.8031	0.8579	0.7572
229	Yang-server	0.8013	0.8334	0.7745
166	RaptorX	0.7989	0.8497	0.7564
288	DFolding-server	0.7969	0.8516	0.7511

282	GuijunLab-Threader	0.7941	0.8516	0.7459
35	Manifold-E	0.7938	0.8406	0.7547
298	MUFold	0.7925	0.8569	0.7386
98	GuijunLab-Assembly	0.7912	0.8571	0.736
383	server_124	0.7905	0.8528	0.7383
188	GuijunLab-DeepDA	0.7904	0.8538	0.7374
125	UltraFold_Server	0.7872	0.8529	0.7321
353	hFold	0.7872	0.8474	0.7368
446	Shennong	0.785	0.858	0.724
462	MultiFOLD	0.7752	0.8546	0.7087
466	ColabFold	0.774	0.8005	0.7517
270	NBIS-AF2-standard	0.7508	0.7918	0.7165

Supplementary Table 6. The comparison between MULTICOM_refine and NBIS-AF2-standard on 65 full-length targets (36 FM targets and 29 TBM targets) in terms of average GDT-TS of top 1 models or best of five models. The p-value for the difference between MULTICOM_refine and NBIS-AF2-standard is calculated by the one-sided Wilcoxon signed rank test.

	Top 1 Model			Best of five Models		
Domain type	MULTICOM_refine	NBIS-AF2-standard	p-value	MULTICOM_refine	NBIS-AF2-standard	p-value
All 65 targets	0.7469	0.7200	0.02137	0.7684	0.7330	2.884e-05
36 FM targets	0.6411	0.6125	0.1481	0.6750	0.6278	0.0006163
29 TBM targets	0.8781	0.8533	0.02788	0.8843	0.8635	0.01226

Supplementary Table 7. The comparison between the average GDT-TS of the original models and the average GDT-TS of the refined models on the 23 single-chain monomer targets. Bold font denotes a higher (or equal) average score.

Target #	Avg GDT-TS of original models	Avg GDT-TS of refined models	Target #	Avg GDT-TS Of original models	Avg GDT-TS of refined models
T1104	**0.78762**	0.78076	T1155	**0.65482**	0.64564
T1112	0.78402	**0.79098**	T1159	**0.97594**	0.97436
T1120	**0.5033**	**0.5033**	T1162	**0.96198**	0.96042
T1122	**0.27074**	0.26596	T1163	**0.96224**	0.95054
T1130	**0.29308**	0.25032	T1175	**0.92834**	0.914

T1131	0.18944	**0.19782**	T1177	0.89196	**0.89418**
T1133	0.94166	**0.94974**	T1180	0.72928	**0.88324**
T1139	0.8756	**0.87626**	T1182	**0.85726**	0.78036
T1145	0.74144	**0.80566**	T1183	**0.96204**	0.95412
T1146	0.94692	**0.94836**	T1188	0.96684	**0.96868**
T1147	**0.99126**	0.9864	T1194	**0.98786**	0.9764
T1150	**0.9371**	0.9345			

Supplementary Table 8. The average GDT-TS of the top 1 model for all 94 domains, 39 domains from 27 single-chain targets, and 55 domains from 41 targets belonging to protein assemblies predicted by our two human predictors (MULTICOM and MULTICOM_human) and four server predictors (MULTICOM_refine, MULTICOM_egnn, MULTICOM_deep, MULTICOM_qa).

Predictor	Avg. GDT-TS on 94 domains	Avg. GDT-TS on 39 domains from 27 single-chain targets	Avg. GDT-TS on 55 domains from 41 targets belonging to assemblies
MULTICOM	**0.8073**	0.7520	**0.8464**
MULTICOM_human	0.8057	0.7520	0.8438
MULTICOM_refine	0.7964	0.7538	0.8266
MULTICOM_egnn	0.793	0.7513	0.8225
MULTICOM_deep	0.7922	**0.7541**	0.8192
MULTICOM_qa	0.7917	0.7438	0.8256

Supplementary Table 9. The ratio of models with correct topology (TM-score > 0.5) in the model pool predicted by the single-chain structure prediction for 29 TBM targets and 36 FM targets. The targets with a low ratio (< 0.1) are highlighted in bond font.

TBM targets				FM and FM/TBM targets			
Target No.	Ratio of correct models	Target No.	Ratio of correct models	Target No.	Ratio of correct models	Target No.	Ratio of correct models
T1106s2	1	T1152	1	T1104	0.93	**T1137s6**	**0.06**
T1109	1	T1153	1	T1106s1	1	T1145	1
T1110	1	T1157s1	1	T1112	1	T1150	1
T1119	1	T1157s2	1	T1113	1	T1151s2	1

T1124	1	T1158	1	T1120	0.94	T1154	0.69
T1127	1	**T1160**	**0**	T1121	1	T1155	1
T1132	1	**T1161**	**0**	**T1122**	**0.02**	T1159	1
T1133	1	T1162	1	T1123	0.9	T1169	0.73
T1134s1	1	T1163	1	**T1125**	**0**	T1173	0.42
T1137s7	1	T1170	1	T1129s2	1	T1174	0.98
T1137s8	1	T1175	1	**T1130**	**0**	T1177	1
T1137s9	1	T1180	1	**T1131**	**0**	T1178	0.83
T1139	1	T1183	1	T1134s2	1	T1179	0.92
T1146	1	T1188	1	**T1137s1**	**0**	T1181	1
T1147	1			**T1137s2**	**0**	T1182	1
				**T1137s3**	**0**	T1184	1
				**T1137s4**	**0**	T1187	1
				T1137s5	0.22	T1194	1
Average			0.9310				0.6846

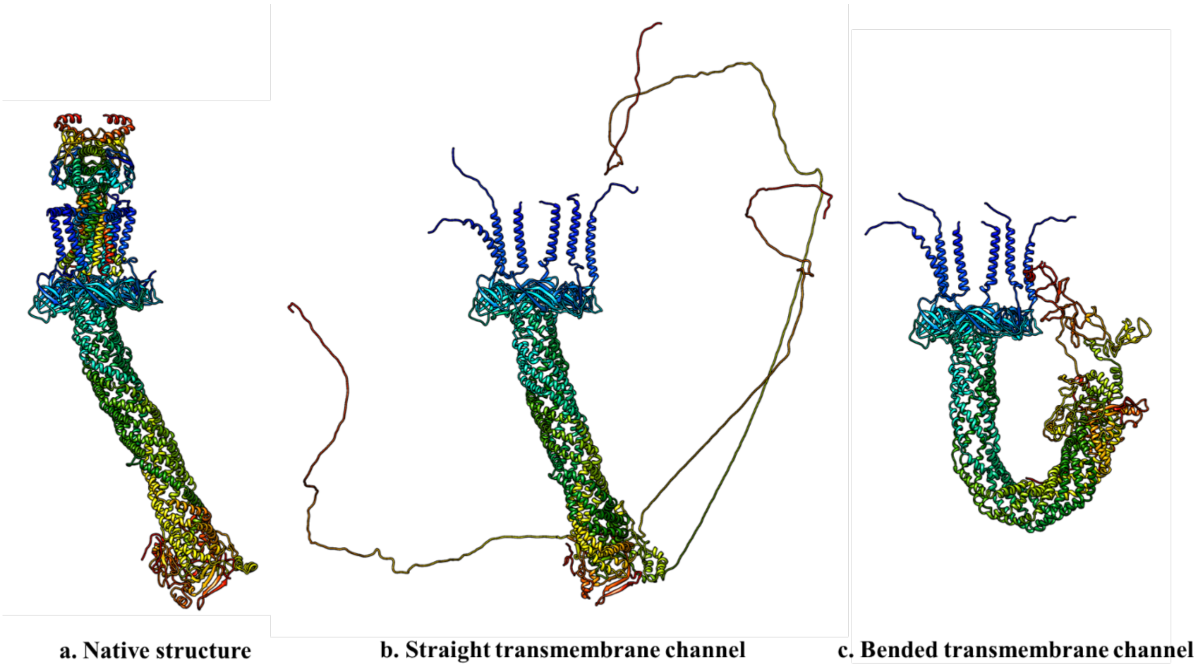

Supplementary Figure 1. (a) The native structure for H1137 consisting of 9 different chains (stoichiometry: A1B1C1D1 E1F1G2H1I1); **(b)** The predicted, straight complex structure (transmembrane channel) formed by the six chains (T1137s1-T1137s6) of H1137; and **(c)** The predicted, bended complex structure for the six chains (T1137s1-T1137s6) of H1137. The long extended coil in (b) and (c) are disordered residues that do not exist in the native structure. It may be useful to trim the disordered residue prior to predict the structure of the six chains as demonstrated by Yang-Server. Overall, the similarity between (b) and (a) is higher than between (c) and (a). Therefore, the tertiary structures of the six chains in (b) are more similar to the native structures than those in (c).

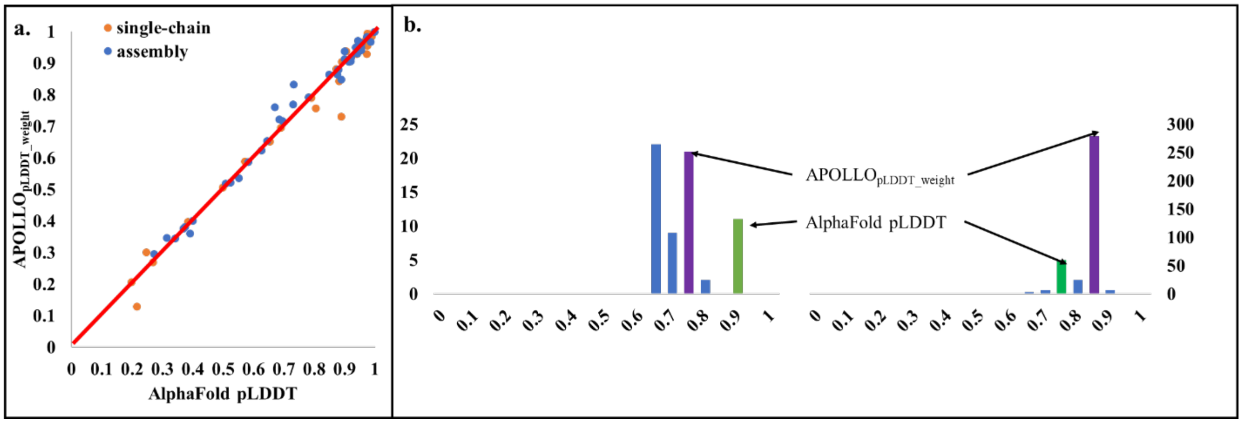

Supplementary Figure 2. (a) The GDT-TS of selected top1 model from AlphaFold2 pLDDT score plotted against that of APOLLO$_{pLDDT_weight}$ on 68 targets of human_model_dataset. **(b)** The distribution of true GDT-TS scores of models for T1180 (left) and T1106s1 (right). The bin from which the top 1 model selected by AlphaFold2 pLDDT and APOLLO$_{pLDDT_weight}$ is highlighted in green and purple, respectively. AlphaFold2 pLDDT selected a good model for T1180 but a bad model for T1106s1, while APOLLO$_{pLDDT_weight}$ selected a good model for T1161s1 but a bad model for T1180.

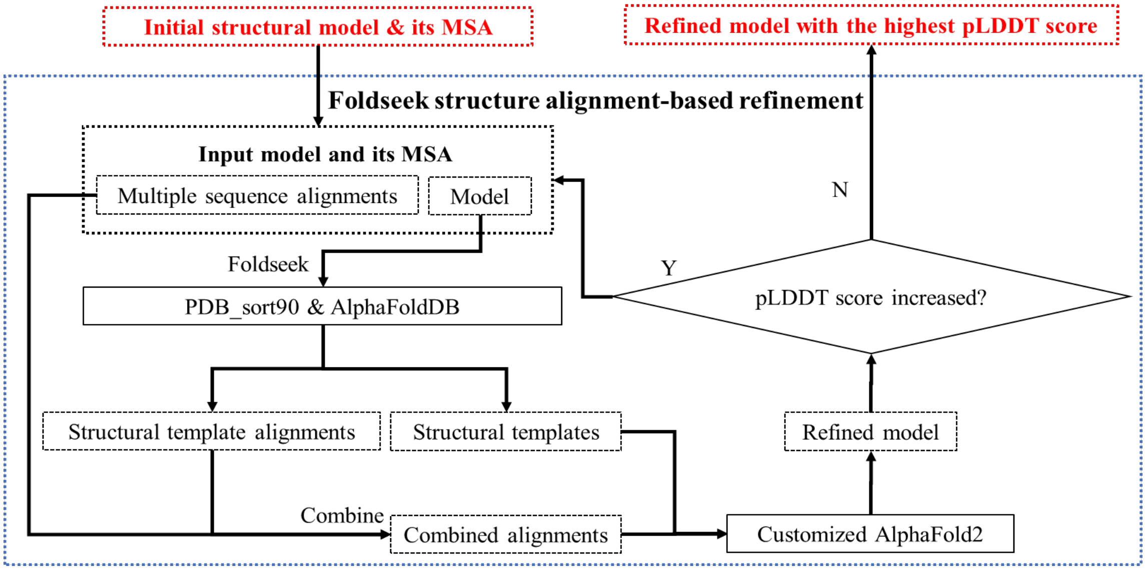

Supplementary Figure 3. The workflow of the Foldseek-based refinement method.
